# Survival from prostate cancer in England and Wales up to 2001

**DOI:** 10.1038/sj.bjc.6604595

**Published:** 2008-09-23

**Authors:** S Rowan, B Rachet, D M Alexe, N Cooper, M P Coleman

**Affiliations:** 1 Office for National Statistics (Room 2000), Segensworth Road, Titchfield, Fareham, Hants PO15 5RR, UK; 2 Cancer Research UK Cancer Survival Group, Non-Communicable Disease Epidemiology Unit, Department of Epidemiology and Population Health, London School of Hygiene and Tropical Medicine, Keppel Street, London WC1E 7HT, UK; 3 Social and Health Analysis and Reporting Division, Office for National Statistics (Room FG/114), 1 Myddelton Street, London EC1R 1UW, UK

Within the last 10 years, prostate cancer has become the most common malignancy among men in England and Wales. The age-standardised incidence rate exceeded that for colorectal cancer by 1993, and overtook the (declining) rate for lung cancer in 1999 (Quinn *et al*, 2001). By the early 2000s, approximately 29 000 men were diagnosed each year with prostate cancer, accounting for one in four of all new cancers in men (excluding nonmelanoma skin cancer). Prostate cancer is rare under the age of 50 years, but incidence rises steeply with age, reaching nearly 1000 cases per 100 000 per year (1% annual risk) in men aged 85 years and more. Each year, there are some 9000 deaths from prostate cancer, approximately one in eight of all cancer deaths in men (Office for National Statistics, 2003).

The causes of prostate cancer are not well known. Family history in first-degree relatives is a risk factor, and incidence is 60% higher in African Americans and 38% lower in Asian Americans than in US Whites (Platz and Giovannucci, 2006), but possible risk factors related to nutrition, environment, lifestyle, sexual history, occupation and ethnicity have not been conclusively identified. The natural history of prostate cancer is poorly understood, ranging from clinically indolent cancers to highly aggressive and often fatal disease (Breslow *et al*, 1977). Treatment in the 1990s included various combinations of surgery, radiotherapy, chemotherapy and endocrine therapy, but early disease was sometimes managed by ‘watchful waiting’, and other modalities, such as cryotherapy, ultrasound and laser treatment have also been used for local disease control (Kirby, 1996a, 1996b).

Age-standardised incidence of prostate cancer rose slowly from 1971 up to the late 1980s, but tripled during the 1990s to more than 90 cases per 100 000 per year. This rapid increase is largely attributable to the increasingly widespread use of prostate-specific antigen (PSA) testing, which has led to an increase in the recorded incidence of localised prostate cancer (Evans and Møller, 2003; Pashayan *et al*, 2006a, 2006b).

Prostate cancer is now more common among the affluent. In the early 1980s, incidence was 35–40 cases per 100 000 per year in all deprivation groups. In the decade up to the mid-1990s, however, incidence increased more than two-fold among the most affluent men, more rapidly than among the most deprived men, and is now about 40% higher in the most affluent groups (Quinn *et al*, 2001). Age-standardised mortality rates remained stable from 1950 to the 1980s before rising gradually, reaching a peak of 31 cases per 100 000 per year in 1993, then declining slightly thereafter.

We analysed the data for over 201 000 men diagnosed with a first, primary, malignant neoplasm of the prostate in England and Wales during the 14-year period 1986–1999 and who were followed up to the end of 2001, some 86% of the 233 000 men potentially eligible for inclusion. Approximately 9% (19 800 men) had a recorded survival of zero, most of them who were registered solely from a death certificate, but for a further 1.4% (3300) the vital status was unknown on 5 November 2002, when the data were extracted for analysis, and 3.2% (7500) of men were excluded because the prostate cancer was not their first primary cancer.

The vast majority of prostate cancers are adenocarcinoma (Ross and Schottenfeld, 1996). The proportion so described in England and Wales rose from just under 60% of all cases in the early 1990s to approximately 85% by the early 2000s, in parallel with a decline in the proportion coded as epithelial malignancy without further specification, from 28 to approximately 10% (data not shown). This pattern suggests steady improvement in the recording of pathological data, rather than a shift in the type of malignancy.

## Survival trends

**Figure 1:**
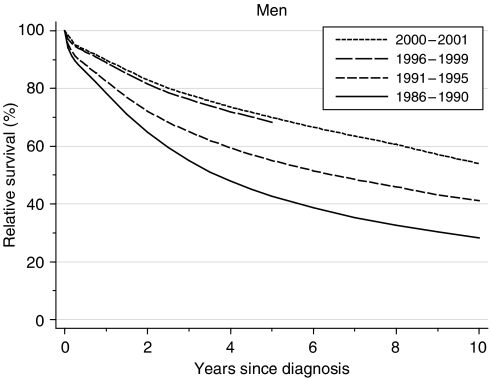
Relative survival (%) up to 10 years after diagnosis by calendar period of diagnosis: England and Wales, adults (15–99 years) diagnosed during 1986–1999 and followed up to 2001. Survival estimated with cohort or complete approach (1986–1990, 1991–1995, 1996–1999) or hybrid approach (2000–2001) (see Rachet *et al*, 2008).

For men diagnosed during 1996–1999, 1-year survival rose to 89%, from 78% a decade or so earlier. This represents a rapid deprivation-adjusted increase of some 6% every 5 years since 1986–1990 (Table 1, Figure 1).

Five-year survival has increased with extraordinary rapidity from 43% for men diagnosed during 1986–1990 to 68% for men diagnosed during 1996–1999, an average deprivation-adjusted increase of almost 16% (95% CI: 14.8–17.0%) every 5 years.

For men diagnosed during 1991–1995 and followed up to 2001, 10-year survival had risen to 41%, a similarly rapid rate of increase.

Hybrid analysis (Brenner and Rachet, 2004) based on the follow-up of survivors during 2000–2001 suggests that survival will continue to improve, but more slowly than over the previous decade, reaching 90% at 1 year and 70% at 5 years (Table 1).

## Deprivation

**Figure 2:**
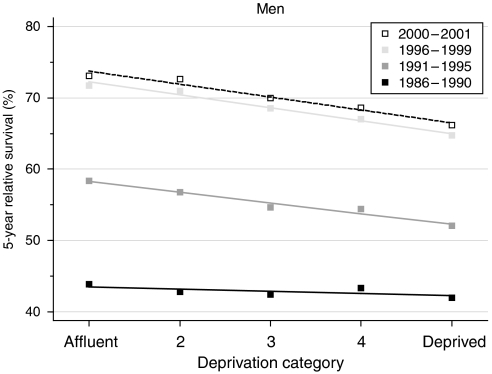
Trends in the deprivation gap in 5-year relative survival (%) by calendar period of diagnosis: England and Wales, adults (15–99 years) diagnosed during 1986–1999 and followed up to 2001.

Despite high overall survival, there is a significant deprivation gap in survival, which increased significantly during the 1990s (Figure 2). Five-year survival for men in the most deprived groups who were diagnosed during 1996–1999 was some 7% lower than for men in the most affluent group (deprivation gap –7.2%). This represents a significant widening of the deprivation gap by an average of −3.2% every 5 years since 1986–1990 (Table 2).

Hybrid analysis based on the follow-up of men who were alive during all or part of the period 2000–2001 suggests that the deprivation gap may not widen any further, with a deprivation gap of −4 to −7% in relative survival at 1, 5 and 10 years (Table 2).

## Comment

The recent trends in prostate cancer survival are remarkable. Survival improved by approximately 5–6% every 5 years between 1971–1975 and 1981–1985, but it did not change at all between the early and late 1980s (Coleman *et al*, 1999). The very rapid increase reported here, represents a sharp reacceleration of survival during the 1990s.

However, the patterns and trends in prostate cancer incidence and survival are not an artefact. They reflect a rapid and substantial shift in the biological and clinical spectrum of prostate cancer, as we now define, diagnose, register and treat it, following the introduction of sensitive new diagnostic techniques such as the PSA test.

The PSA test enables invasive prostate cancer to be identified earlier than it might have been diagnosed clinically on the basis of symptoms, but the test also enables the identification of latent tumours that may never have caused symptoms during the man's lifetime (Horwich *et al*, 1995). Thus, the chance of a man in England and Wales being diagnosed with prostate cancer between his sixtieth and eightieth birthdays rose from 4%, based on the pattern of incidence by age throughout the 1980s, to just 5% by 1990, but it almost doubled over the next 10 years to reach 9% by 2000 (Table 3). At a population level, therefore, prostate cancer as defined in the PSA era since the early 1990s is no longer the same disease as it was in the 1980s.

In the absence of widespread access to a substantially improved treatment for early prostate cancer in the last 15 years of the 20th century, it seems most likely that the extremely rapid improvement in recorded survival for men diagnosed with prostate cancer during the period 1986–1999 reflects an increase in the diagnosis, treatment and registration of men with asymptomatic malignancy as a result of the increasingly widespread use of PSA tests during the 1990s (Evans and Møller, 2003).

The concurrent increase in the difference in survival between affluent and deprived men, taken with the increase in incidence, similarly more rapid among men in affluent groups, also suggests that affluent men have had greater access to PSA tests than men in the more deprived groups.

A minority of cases (8.5%) were excluded from analysis because the only available information was from a death certificate; hence, their duration of survival was unknown. These men probably have had shorter survival than the average for men who were registered during their lifetime (Berrino *et al*, 1995). The proportion of such cases differed very little among deprivation categories or over time; however, (data not shown), so their exclusion is most unlikely to have biased the estimates of the widening deprivation gap in survival.

In Europe, the international range in 5-year survival for men diagnosed with prostate cancer during 1990–1994 was wider than for any other cancer, ranging from 39% in Poland to 85% in Austria (Sant *et al*, 2003). Survival in the Nordic countries was high, and rose further during the 12 years up to 1994, with the exception of Denmark, where survival was lower and barely increased at all (Coleman *et al*, 2003). As with the socioeconomic differences in the incidence and survival trends reported here, these patterns seem likely to be at least in part attributable to international differences in the intensity of diagnostic and screening activity.

The implications for asymptomatic men diagnosed with prostate cancer following a PSA test are important. The randomised trials of mass screening for prostate cancer with the PSA test are not expected to report mortality results until 2010 (de Koning *et al*, 2002; Hakama *et al*, 2008).

## Figures and Tables

**Table 1:** Trends in relative survival (%) by time since diagnosis and calendar period of diagnosis: England and Wales, men (15–99 years) diagnosed during 1986–1999 and followed up to 2001

		**Calendar period of diagnosis^a^**						**Average change (%)**		**Prediction^c^ for patients**	
		**1986–1990**		**1991–1995**		**1996–1999**		**every 5 years^b^**		**diagnosed during 2000–2001**	
**Time since diagnosis**		**Survival (%)**	**95% CI**	**Survival (%)**	**95% CI**	**Survival (%)**	**95% CI**	**Survival (%)**	**95% CI**	**Survival (%)**	**95% CI**
1 year	Men	**78.3**	(77.9, 78.7)	**82.5**	(82.2, 82.8)	**88.9**	(88.6, 89.2)	6.2**	(5.5, 6.8)	**89.9**	(89.5, 90.3)
5 years	Men	**42.7**	(42.1, 43.3)	**55.1**	(54.6, 55.6)	**68.4**	(67.7, 69.0)	15.9**	(14.8, 17.0)	**69.9**	(69.2, 70.6)
10 years	Men	**28.2**	(27.5, 28.8)	**41.1**	(40.2, 41.9)			16.4**	(13.8, 19.0)	**54.0**	(52.8, 55.2)

CI=confidence interval.

a Survival estimated with cohort or complete approach (see Rachet *et al*, 2008).

b Mean absolute change (%) in survival every 5 years, adjusted for deprivation (see Rachet *et al*, 2008).

c Survival estimated with hybrid approach (see Rachet *et al*, 2008).

** *P*<0.01.

**Table 2:** Trends in the deprivation gap in relative survival (%) by time since diagnosis and calendar period of diagnosis: England and Wales, men (15–99 years) diagnosed during 1986–1999 and followed up to 2001

		**Calendar period of diagnosis^a^**						**Average change (%)**		**Prediction^c^ for patients**	
		**1986–1990**		**1991–1995**		**1996–1999**		**every 5 years^b^**		**diagnosed during 2000–2001**	
**Time since diagnosis**		**Deprivation gap (%)**	**95% CI**	**Deprivation gap (%)**	**95% CI**	**Deprivation gap (%)**	**95% CI**	**Deprivation gap (%)**	**95% CI**	**Deprivation gap (%)**	**95% CI**
1 year	Men	−3.0**	(−4.2, −1.8)	−4.2**	(−5.1, −3.3)	−4.1**	(−4.9, −3.3)	**−0.6**	(−1.3, 0.2)	−4.4**	(−5.5, −3.3)
5 years	Men	**−1.2**	(−2.9, 0.4)	−6.0**	(−7.4, −4.6)	−7.2**	(−9.0, −5.5)	−3.2**	(−4.5, −2.0)	−7.3**	(−9.2, −5.3)
10 years	Men	**0.0**	(−1.9, 1.8)	−4.9**	(−7.3, −2.5)			−4.9**	(−7.9, −1.8)	−6.7**	(−9.9, −3.6)

CI=confidence interval.

a Survival estimated with cohort or complete approach (see Rachet *et al*, 2008).

b Mean absolute change (%) in the deprivation gap in survival every 5 years, adjusted for the underlying trend in survival (see Rachet *et al*, 2008).

c Survival estimated with hybrid approach (see Rachet *et al*, 2008).

** *P*<0.01.

**Table 3:** Incidence per 100 000 per year and cumulative risk (%) between sixtieth and eightieth birthdays, England and Wales, 1982–2000


	**Year of diagnosis**				
**Age (years)**	**1982**	**1985**	**1990**	**1995**	**2000**
60–64	57	65	96	129	202
65–69	130	130	172	253	384
70–74	226	233	292	416	539
75–79	375	382	487	610	706
					
Cumulative risk (%)	3.86	3.97	5.10	6.80	8.75
